# Prediction of Input–Output Characteristic Curves of Hydraulic Cylinders Based on Three-Layer BP Neural Network

**DOI:** 10.3390/s25061949

**Published:** 2025-03-20

**Authors:** Wei Cai, Yirui Zhang, Jianxin Zhang, Shunshun Guo, Rui Guo

**Affiliations:** 1 State Key Laboratory of Crane Technology, Yanshan University, Qinhuangdao 066004, China; guorui@ysu.edu.cn; 2 School of Mechanical Engineering, Yanshan University, Qinhuangdao 066004, China; zhangyirui@stumail.ysu.edu.cn (Y.Z.);; 3 Hebei Provincial Key Laboratory of Heavy Machinery Fluid Power Transmission and Control, Yanshan University, Qinhuangdao 066004, China

**Keywords:** hydraulic cylinder, displacement control, prediction model, BP neural network

## Abstract

To predict the variation in the displacement position of hydraulic cylinder piston rods, a neural network model is proposed to enhance the displacement control accuracy of hydraulic cylinders. The innovation of this paper is that by calculating the compressibility-induced flow loss of hydraulic fluid, mathematical models for both the internal and external leakage of hydraulic cylinders are established, identifying seven primary factors influencing piston rod displacement. Because there are many influencing factors and complex parameters different from traditional backpropagation (BP) neural network used in previous studies, this paper innovatively proposes a three-layer BP neural network ensemble model for predicting input–output characteristic curves of hydraulic cylinders. In the process of model improvement, a nonlinear adaptive decreasing weight mechanism is introduced to improve the optimization accuracy of the algorithm, facilitating the search for optimal solutions. The most reasonable weight and bias parameters are determined via the iterative training and testing of each BP neural network layer. This model enables the real-time prediction of piston rod displacement output curves after a specified time interval based on external input parameters. The predicted time is utilized to compensate for the response delays caused by directional valve switching and hydraulic fluid buffering, thereby enabling proactive displacement prediction. Validation results demonstrate that the maximum predicted displacement error is reduced to 0.5491 µm, with the model’s maximum runtime being 27.82 ms. The maximum allowable time allocated for directional valve switching and fluid buffering in the hydraulic system is extended to 74.57 ms, achieving the objective of enhancing both displacement control accuracy and operational efficiency.

## 1. Introduction

Remarkable advancements in electrohydraulic control technologies [1] have substantially diversified the functional capabilities of hydraulic systems [2] in the construction machinery domain. High-precision actuation and superior operational reliability [3] now represent pivotal developmental trajectories in hydraulic technology. The automatic monitoring and diagnosis of system parameters such as flow, pressure, temperature, and oil contamination [4] using electronic components such as sensors supplemented by systematic data analyses and mining by computer systems [5] can significantly improve the control accuracy and operation efficiency of hydraulic systems.

Notably, displacement control precision currently constitutes one of the most stringent performance metrics in high-end hydraulic systems. As the predominant actuation component in such systems [6], the displacement accuracy of hydraulic cylinder piston rods has a deterministic influence on overall system controllability and operational efficacy [7]. Consequently, rigorous investigation into output displacement calibration methodologies for hydraulic cylinders are of paramount importance for advancing precision control paradigms in modern hydraulic systems. The hydraulic cylinder displacement sensor is a critical component of hydraulic systems, designed to accurately measure the displacement of the hydraulic cylinder piston [8]. It primarily encompasses technologies such as magnetostrictive, inductive, capacitive, ultrasonic, and fiber-optic sensors [9]. Despite significant advancements in hydraulic cylinder displacement sensor technology in recent years, the precision of piston rod displacement remains influenced by a multitude of factors, including pressure, flow rate, temperature, hydraulic oil viscosity, density, compressibility, and leakage [10]. Additionally, conventional approaches continue to demonstrate constrained efficacy in meeting the stringent requirements of control accuracy and transient response dynamics. To address these limitations, Wei et al. [11] analyzed the dynamic characteristics of a digital hydraulic cylinder, and they established a mathematical model of the equivalent flow area of the valve port of the primary and secondary throttling slots to allow for an analysis of the initial position of the piston. Ding et al. [12] proposed a hydraulic cylinder system model with three conversion modes to expand the displacement tracking capability of hydraulic cylinders. Kim et al. [13] proposed an adaptive backstepping control (ABSC) scheme to overcome the uncertainty in hydraulic cylinder systems and improve their tracking performance. Batabyal et al. [14] proposed an automatic control method for the mechanical operating position of hydraulic cylinders under digital twins, and it showed that the steady-state displacement error was small under both constant and variable load conditions. Zhang et al. [15] proposed a hydraulic cylinder system model based on a compound velocity feedforward and fuzzy proportional integral derivative (PID) control strategy, achieving a displacement tracking error of 3%. Ding et al. [16] proposed a least mean square (LMS) adaptive filter based on wavelet transform, which has strong ability to track the displacement of the piston rod of hydraulic cylinders. Zhang et al. [17] studied the mechanism of a fuzzy PID controller to improve displacement tracking response performance and realized running speed control. Lin et al. [18] proposed a design method for robust discrete-time sliding mode control, which was experimentally verified to be better in tracking accuracy than PID control. Yan et al. [19] proposed an improved continuous electrohydraulic servo model based on synovium control to solve the problem of buffeting when the electrohydraulic servo system is moving to a near-zero position and tracking the displacement trajectory of the piston rod. Ahn et al. [20] proposed a displacement control method based on an adaptive reverse-control scheme, which also has relatively good tracking accuracy in the case of external interference. Wu et al. [21] proposed an electrohydraulic servo system controlled by the displacement and force coupling of a bidirectional hydraulic press, and they established a corresponding mathematical model that ultimately reduced the overshoot of displacement and increased the degree of freedom of the system.

Although the aforementioned designs of hydraulic cylinder piston rod displacement systems integrate multiple high-precision technologies, such as automatic control, adaptive filtering, and digital twins, their complexity in monitoring and control, coupled with prolonged response times, hinders the accurate acquisition of positional changes in mechanical operational behavior. This presents a significant challenge in enhancing the displacement accuracy of hydraulic cylinders. In contrast to traditional methods such as hydraulic servo closed-loop control, the application of neural networks for predicting hydraulic cylinder input–output curves offers distinct advantages. Neural networks leverage data-driven approaches to simplify control architectures, capture the nonlinear characteristics of hydraulic systems, and exhibit adaptive and real-time predictive capabilities, thereby addressing the limitations of conventional techniques.

Because the traditional BP neural network prediction method does not involve many influential factors, its structure is relatively simple. Differently from previous methods, in this study, we focus on the input–output curves of hydraulic cylinders and address the challenge of controlling displacement accuracy by a three-layer BP neural network based on nonlinear adaptive descending weight to enhance the precision of hydraulic cylinder piston rod displacement. We primarily analyze the factors influencing conventional hydraulic cylinders, such as the effective bulk modulus of the fluid, viscosity, temperature, pressure, flow rate, and contaminants, to identify the parameters that need to be collected under various conditions and operational scenarios. Such parameters include the physical properties of the hydraulic fluid and piston rod displacement. By utilizing the model to predict hydraulic cylinder displacement, the system can anticipate the piston rod’s position at different time points, enabling the early closure of directional valves. This approach compensates for the response time of hydraulic components, thereby improving displacement accuracy and overcoming the inherent delays associated with traditional displacement compensation methods. Automatic parameter optimization in the neural network prediction process is realized. The proposed model demonstrates significant practical value in addressing the issue of fluid motion latency in hydraulic systems.

This paper is organized as follows. Section 1 presents the introduction. In Section 2, we formulate corresponding models for hydraulic cylinders. Building upon the models developed in the preceding section, in Section 3, we design a predictive model for the input and output of hydraulic cylinders. In Section 4, we validate and optimize the predictive model. Finally, Section 5 presents the conclusions.

## 2. Establishment of Hydraulic Cylinder-Related Model

### 2.1. Oil Expansion and Contraction Loss

#### 2.1.1. Compressible Flow Loss Model

**Figure 1:**
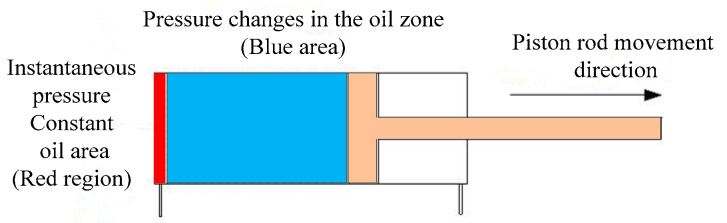
Non-rod chamber oil pressure division area schematic diagram.

To illustrate the pressure distribution within the non-rod chamber during the extension motion of a double-acting single-piston-rod hydraulic cylinder, a schematic diagram is presented in Figure 1. The red region represents the hydraulic fluid that instantaneously enters the non-rod chamber. Due to the extremely short time interval, the pressure in this region can be assumed to remain constant. During the extension of the piston rod, external loads typically exhibit fluctuations or even significant variations, causing the hydraulic fluid in the blue region of the non-rod chamber to undergo compression or expansion. This results in displacement errors in the motion of the piston rod. Therefore, it is necessary to establish a mathematical model for the volume of fluid compressed or expanded per unit time and analyze it precisely. To facilitate calculations, the displacement error of the piston rod caused by fluid compressibility is quantified in terms of flow loss.

When the pressure of a liquid changes, its volume changes, and this property is called compressibility, which is described with β, as shown in Equation (1):

(1) $$\beta =-\frac{\Delta V_{\beta }}{V_{\beta }\Delta p_{\beta }}$$

Here $V_{\beta }$ is the volume of the fluid, $\Delta V_{\beta }$ is the decrease in volume, and $\Delta p_{\beta }$ is the increase in pressure.

The reciprocal of the compression coefficient is called the bulk elastic modulus [22], as shown in Equation (2):

(2) $$\begin{array}{c} E=\frac{1}{\beta }=-\frac{V_{\beta }\Delta p_{\beta }}{\Delta V_{\beta }} \end{array}$$

It is assumed that the bottom area of the double-acting single piston rod is $S_{0}$. At time $t_{0}$, the movement distance of the piston rod is $x_{0}$. At time $t_{0}+\Delta t$, the pressure change is $\Delta p_{1}$, the distance change of the piston rod is Δx, time Δt is very small, and the pressure and temperature of the oil do not change much. It is considered that the effective bulk elastic modulus of the oil during this period is a fixed value, that is, $E_{e}$. During this time interval, the oil flow loss of the non-rod chamber of the hydraulic cylinder is $Q_{0}$, as shown in Equation (3):

(3) $$Q_{0}=\frac{\Delta V_{\beta }}{\Delta t}=\frac{S_{0}\left(x_{0}+\Delta x\right)\Delta p_{1}}{E_{e}\Delta t}$$

If $x_{0}+\Delta x=x_{1}$, $\frac{\Delta p_{1}}{\Delta t}=\dot{p}$ is set, the equation for the flow loss becomes:

(4) $$Q_{0}=\frac{S_{0}x_{1}}{E_{e}}\dot{p}$$

Here, $\dot{p}$ is the oil pressure change rate of the non-rod chamber. $S_{0}$ is a constant value, $E_{e}$ is proportional to the pressure of the oil and inversely proportional to the temperature, so $Q_{0}$ is mainly affected by the displacement of the piston rod, non-rod chamber oil pressure change rate, oil pressure, and temperature.

#### 2.1.2. Thermal Expansion Flow Loss Model

Thermal expansibility is a property referring to an increase in the temperature and volume of liquid [23], and it is represented by $\alpha_{V}$, as shown in Equation (5):

(5) $$\alpha_{V}=\frac{1}{V_{\alpha }}\frac{\Delta V_{\alpha }}{\Delta T_{\alpha }}$$

Here, $\Delta T_{\alpha }$ is the change in the liquid temperature and $V_{\alpha }$ is the change in the liquid volume.

The expansion coefficient of liquid varies little under pressure, and it is believed that it depends only on the temperature of the liquid [24]. The external temperature is set to 298 K (25 °C), and the expansion coefficient of the L-HL hydraulic oil [25] is 6.2 × 10^−4^ 1/K. It is supposed that the value in Equation (5) is 1 K after derivation and calculation, as shown in Equation (6):

(6) $$6.2\times 10^{-4}=\frac{1}{V_{\alpha }}\frac{\Delta V_{\alpha }}{\Delta T_{\alpha }}=\frac{1\times S_{0}\Delta x}{S_{0}x_{0}\times 1}=\frac{\Delta x}{x_{0}}$$

For the hydraulic oil in the hydraulic cylinder, the volume of oil lost or gained due to thermal expansion can be negligible.

### 2.2. Hydraulic Cylinder Leakage

#### 2.2.1. Effect of Working Environment on Dynamic Viscosity

The dynamic viscosity of oil is significantly affected by temperature change. The dynamic viscosity value decreases with an increase in temperature [26] and the hydrodynamic viscosity changes with temperature, as shown in Equation (7):

(7) $$\mu_{t}=\mu_{0}e^{-\lambda (T-T_{0})}\lambda$$

Here, $\mu_{t}$ is the dynamic viscosity when the temperature is T °C, $\mu_{0}$ is the dynamic viscosity when the temperature is $T_{0}$ °C [27], and λ is the viscose-temperature coefficient. For hydraulic oil, this can be 0.018–0.036 (1/°C), and 0.025 is taken in this study. The unit of $\mu_{t}$ and $\mu_{0}$ is Pa⋅s.

High pressure has a large effect on the viscosity of hydraulic oil, while low pressure has less of an effect [28]. The viscosity of the oil fluid varies with the pressure, as shown in Equation (8):

(8) $$\mu_{p}=\mu_{1}e^{\alpha p}$$

Here, $\mu_{p}$ is the viscosity when the pressure is p, $\mu_{1}$ is the viscosity when the pressure is 0.1 MPa, and α is the viscosity coefficient, the size of which depends on the physical properties of the liquid and can be approximated as (2–3) × 10^−8^ Pa [29]. The unit of $\mu_{1}$ and $\mu_{p}$ is Pa⋅s.

The amount of air in the oil depends on the nature of the oil, the degree of contact between the oil and the air, and the disturbance state [30]. An increase in the amount of air mixed into the oil will leads to a sharp decline in the effective bulk elastic modulus coefficient of the oil [31]. The dynamic viscosity of the oil increases linearly according to Equation (9):

(9) $$\mu_{B}=\mu_{2}(1+0.015B)$$

Here, B is the percentage of mixed air, $\mu_{B}$ is the dynamic viscosity of the oil when the proportion of mixed air is B, and $\mu_{2}$ is the dynamic viscosity of the oil without mixing air, which is a fixed value.

Based on the empirical formula of the influence of the oil pressure, temperature, and gas amount on the dynamic viscosity, the dynamic viscosity of the oil in the actual pipeline can be obtained, as shown in Equation (10):

(10) $$\mu_{C}=\mu_{3}(1+0.015B)\lambda e^{\alpha p-\lambda (T-T_{0})}$$

Here, $\mu_{C}$ is the dynamic viscosity of the oil with pressure p, temperature T, and mixed gas amount B; $\mu_{3}$ is the pure oil dynamic viscosity when the pressure is 101.03 KPa, the temperature is $T_{0}$, and no gas is mixed; and λ is the viscosity temperature coefficient, which can be 0.018–0.036 (1/°C) for hydraulic oil [32]. The unit of $\mu_{C}$ and $\mu_{3}$ is Pa⋅s.

The influence of the change in the dynamic viscosity of the hydraulic oil on leakage is simplified into coefficient factor $K_{a}$, as shown in Equation (11):

(11) $$K_{a}=\frac{1}{\mu_{3}(1+0.015B)}\lambda e^{\left(\lambda (T-T_{0})-\alpha p\right)}$$

In summary, the coefficient factor $K_{a}$ is primarily influenced by the temperature T and pressure p of the hydraulic fluid in the non-rod chamber at different time points. As $T_{0}$ is a fixed value, the neural network utilizes either $T-T_{0}$ or T to predict the specific value of $K_{a}$.

#### 2.2.2. Hydraulic Cylinder Internal and External Leakage Model

**Figure 2:**
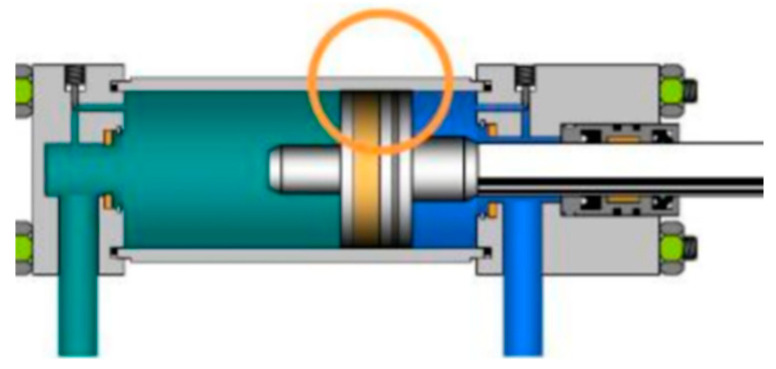
Hydraulic cylinder leakage position diagram.

As illustrated in Figure 2, internal leakage in the hydraulic cylinders refers to the process by which hydraulic oil flows from the high-pressure region to the low-pressure region through the gap between the piston and the cylinder wall [33]. However, the causes of internal leakage in hydraulic cylinders are multifaceted, including factors such as the wear, aging, and deformation of sealing components; the contamination of the hydraulic oil by gases, water, and particulate matter; the rationality of the structural design, and the precision of component machining and assembly [34].

During the operation of a double-acting single-piston-rod hydraulic cylinder, the pressure within the internal chambers typically exhibits an initial gradual increase, followed by a period of stability or continuous variation. Suspended air bubbles or vapor chambers in the hydraulic fluid significantly influence the effective bulk modulus of the liquid [35]. Thus, the effective bulk modulus $E_{B}$ is employed to calculate the leakage volume as a function of pressure changes.

It is assumed that upon the occurrence of leakage, the temperature and pressure between the air bubbles and the hydraulic oil have reached equilibrium. The impact of hydraulic oil pressure changes on its volume can be calculated using the following empirical Equation (12):

(12) $$q_{p}=q_{0}(1+\frac{p_{a}-p_{b}}{E_{B}})$$

Here, $q_{p}$ is the amount of oil leakage when considering the influence of pressure,$q_{0}$ is the theoretical amount of oil leakage without considering the influence of pressure, $p_{a}$ is the pressure value after the pressure change, $p_{b}$ is the pressure value before the pressure change, and $E_{B}$ is the effective bulk elastic modulus of the oil with air proportion B.

Based on Equations (11) and (12), a mathematical model for the internal leakage flow of the hydraulic cylinders can be derived, as shown in Equation (13):

(13) $$Q_{1}=\frac{K_{s}(p_{n}-p_{r})}{\mu_{3}(1+0.015B)}\lambda e^{\left(\lambda (T_{t}-T_{i})-\alpha p_{n}\right)}(1+\frac{\left(p_{n}-p_{r}\right)}{E_{B}})$$

Here, $Q_{1}$ is the internal leakage flow of the hydraulic cylinder when the temperature is $T_{t}$ and the mixed air concentration is B. $K_{s}$ is the structural coefficient of the hydraulic cylinder gap leakage, $p_{n}$ is the pressure of the non-rod chamber, $p_{r}$ is the pressure of the rod end chamber, and $T_{i}$ is the initial temperature of the hydraulic oil.

The internal leakage coefficient of the hydraulic cylinder is $K_{1}$ and the pressure difference between the two chambers of the hydraulic cylinder is $\Delta p_{q}$; thus, the mathematical model of the internal leakage of the hydraulic cylinder can be simplified to Equation (14):

(14) $$Q_{1}=K_{1}\Delta p_{q1}$$

The physical quantities that affect the leakage of the hydraulic cylinder are the non-rod chamber pressure $p_{n}$, rod end chamber pressure $p_{r}$ and temperature $T_{t}$.

The external leakage of the hydraulic cylinder generally comes from the two oil ports and the gap between the piston rod and the inner wall of the cylinder; thus, the external leakage mathematical model of the hydraulic cylinder only considers the external leakage oil flow loss caused by the oil outlet, the piston rod, and the inner wall [36]. The mathematical model is shown in Equation (15):

(15) $$Q_{2}=K_{2}p_{q2}$$

Here, $Q_{2}$ is the external leakage flow loss of the hydraulic cylinder and $K_{2}$ is the external leakage coefficient of the hydraulic cylinder.

The physical factors that affect the external leakage of the hydraulic cylinder are the pressure $p_{r}$ and temperature $T_{t}$ of the rod end chamber.

#### 2.2.3. Hydraulic Cylinder Displacement Flow Model

Taking the extension action of the hydraulic cylinder of the double-acting single piston rod as the research object, a flow–displacement equation is established. The positive direction of the shaft is taken as the direction of the piston rod extension, and the initial position of the piston rod when it is not working is taken as zero to establish the number axis. A flow–displacement equation of the non-rod chamber of the differential cylinder is obtained, as shown in Equation (16):

(16) $$q_{A}-Q_{1}-Q_{2}-\frac{S_{0}x_{1}}{E_{B}}\ddot{p}=S_{0}\dot{x}$$

Here, $q_{A}$ is the flow rate of the non-rod chamber oil inlet of the differential cylinder and $\dot{x}$ is the speed of the piston rod.

We set $\dot{x}=x_{1}-x_{0}/t_{1}-t_{0}$ and $\ddot{p}=p_{n}-p_{r}/t_{1}-t_{0}$, and we substituted the mathematical models of $Q_{1}$ and $Q_{2}$ together into Equation (16). After calculation, Equation (17) is obtained:

(17) $$x_{1}=\frac{E_{B}[(t_{1}-t_{0})(q_{A}-K_{1}\Delta p_{q1}-K_{2}p_{q2})+S_{0}x_{0}]}{S_{0}E_{B}+S_{0}(p_{n}-p_{r})}$$

In Equation (17), the seven physical parameters that affect the displacement of the piston rod of the hydraulic cylinder are the pressure of the non-rod chamber at time $t_{1}$, the pressure of the rod end chamber, the pressure difference between the two chambers, the temperature of the non-rod chamber, the oil flow rate and pressure difference of the non-rod chamber at times $t_{1}$ and $t_{0}$, and the displacement at time $t_{0}$.

## 3. Hydraulic Cylinder Input and Output Prediction Model

### 3.1. Data Acquisition

In the experimental setup, the pressure in the hydraulic cylinder is determined by the external load. Therefore, the external load acting on the piston rod must be continuously varied during the experiment. Additionally, it is essential to ensure that the direction of the external load remains aligned with the central axis of the piston rod in order to avoid experimental errors. To achieve this, a secondary hydraulic system equipped with a pressure-regulating circuit is designed. In this configuration, the piston rod of the secondary hydraulic cylinder is positioned opposite to that of the experimental hydraulic cylinder. By adjusting the set pressure of the relief valve in the pressure-regulating circuit, the system pressure can be modulated, thereby enabling precise and flexible control over the magnitude of the external load applied to the experimental hydraulic cylinder.

In the experimental data measurement process, five sensors were used: two MIK-P300 pressure sensors, one DN6 turbine flow sensor, one pull rope displacement sensor, and one KX-WZP-131 temperature sensor. Data were collected and processed using a data acquisition card and the data acquisition management software DMC, version 1.0.

**Figure 3:**
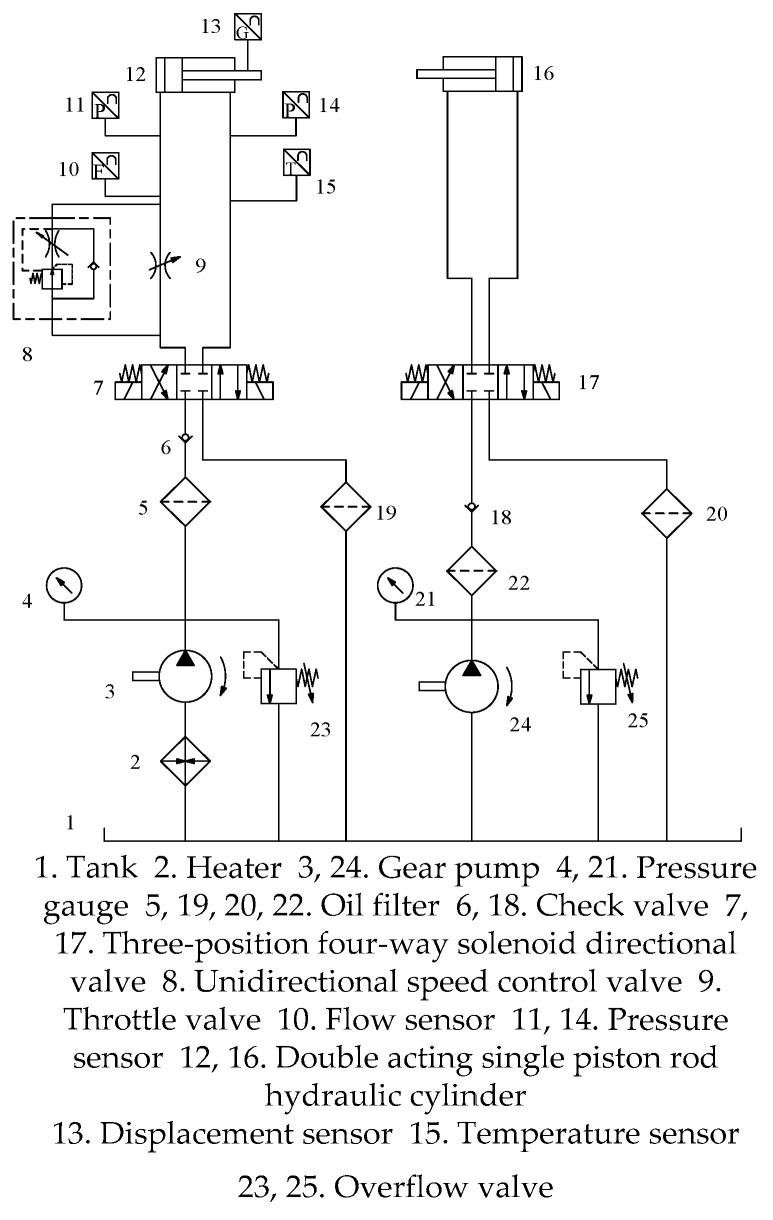
Experimental hydraulic system schematic diagram.

The experimental time of day and the time interval of each experiment should be strictly controlled to prevent different indoor temperatures and system heating from interfering with the hydraulic oil temperature in the experiment. Based on the principal diagram of the experimental hydraulic system shown in Figure 3, the specific experimental steps are as follows:

(1) Check each component of the system, open valves 8 and 9, make sure that each sensor and valve connector are reliably connected without being loose, and make sure that the hydraulic cylinder is in the back state.
(2) Release the two overflow valves, start hydraulic pump 3, observe pressure gauges 4 and 21, adjust overflow valves 23 and 25, adjust the pressure to 12 MPa, and adjust valves 7 and 17 so that the two hydraulic cylinders extend and redraft several times. This is carried out to inspect the system and ensure that the hydraulic chamber is filled with hydraulic oil.
(3) Connect the DC power supply to each sensor, connect the data acquisition system, twist the knob of overflow valve 23, set its pressure to 12 MPa through pressure gauge 4, control the opening degree of reversing valve 7 and throttle valve 9, and check whether the sensor works normally through the DMC interface feedback information.
(4) Determine whether hydraulic cylinder 12 is retractable, adjust pressure gauge 21 to adjust overflow valve 25 to 1.5 MPa, and control reversing valve 17 so that the piston rod of hydraulic cylinder 16 is extended until the piston rod of hydraulic cylinder 12.
(5) Reversing valve 7 extends hydraulic cylinder 12, controls throttle valve 9, makes the flow rate of the hydraulic cylinder oil inlet reach 6 L/min, and records the pressure, flow, temperature, and displacement data.
(6) When hydraulic cylinder 12 completely stops moving, after completing the whole process, control reversing valve 7 so that hydraulic cylinder 12 can quickly return through the check valve element of the speed control valve.
(7) Adjust the pressure of overflow valve 25 to 2 MPa, repeat steps (4), (5), and (6), and keep the flow rate stable at 6 L/min by adjusting the button of the speed control valve during the experiment.
(8) Repeat step (7) and increase the pressure by 1 MPa each time by adjusting the knob of overflow valve 25. At the same time, fine-tune the knob of the speed control valve to maintain the stability of the hydraulic cylinder oil inlet flow.
(9) Through the above experimental steps, experimental data under the same temperature, the same flow, and different pressures can be obtained. Repeat the experiment continuously by following these 8 test steps, and increase the pressure of the relief valve 25 by 0.5 MPa in each test until the pressure data collected reaches 10 MPa.
(10) Adjust throttle valve 9, reduce the flow rate of the oil inlet of the hydraulic cylinder by 1 L/min each time, repeat the above 8 experimental steps, and record the data at the same time. When the flow rate is reduced to less than 3 L/min, close throttle valve 9 and use only speed control valve 8 to control the speed of the hydraulic cylinder.

During the above experimental process, keep the oil temperature of the system stable. After all 10 steps are completed, use heater 2 to increase the oil temperature, ensure that the oil temperature rises by 1 °C each time, and continue to record the test data until the temperature reaches 53 °C.

**Figure 4:**
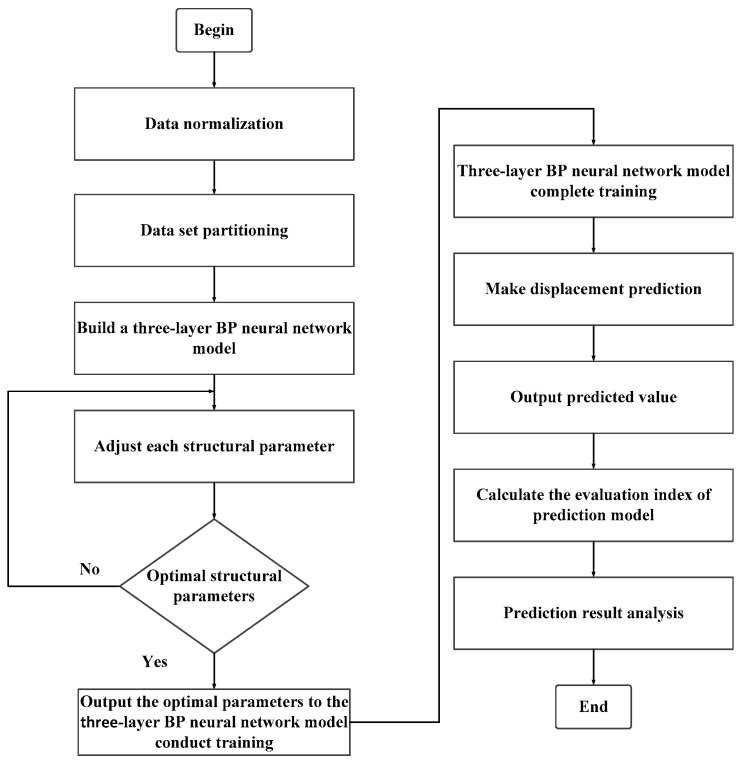
Flowchart of three-layer BP neural network prediction process.

After following the above experimental steps, the experiment is completed, and a displacement dataset under different pressures, flow rates, and temperatures is obtained. After integrating the experimental data, 2500 sets of data were finally obtained, with 42 tables in each group. The table data include the sensor signal data of the non-rod chamber pressure, rod end chamber pressure, hydraulic oil inlet flow rate, temperature, and displacement. The data were subjected to a series of preprocessing steps, which included the imputation of missing values, the detection and treatment of outliers, and the removal of duplicate entries. These data cleansing procedures were implemented to ensure the accuracy and consistency of the dataset. Subsequently, data normalization was carried out, scaling the data to a uniform numerical range in order to eliminate the dimensional disparities among different features, thereby enhancing the model’s learning efficiency and predictive accuracy. Ultimately, the dataset was partitioned into three subsets in a 6:2:2 ratio, designated as the training, validation, and test sets, respectively. A comprehensive flowchart delineating the predictive process of the three-layer BP neural network is presented in Figure 4.

### 3.2. Model Structure Design

In practical applications, most flow sensors have a certain signal delay. The sensor structure, the signal processing circuit, and other factors may cause signal delays [37]. In practical applications, it is necessary to predict the flow value at the starting time point. According to the principle of the predicted displacement and the reason for the flow sensor delay, the internal structure of the predicted model is a nested three-layer BP neural network.

**Figure 5:**
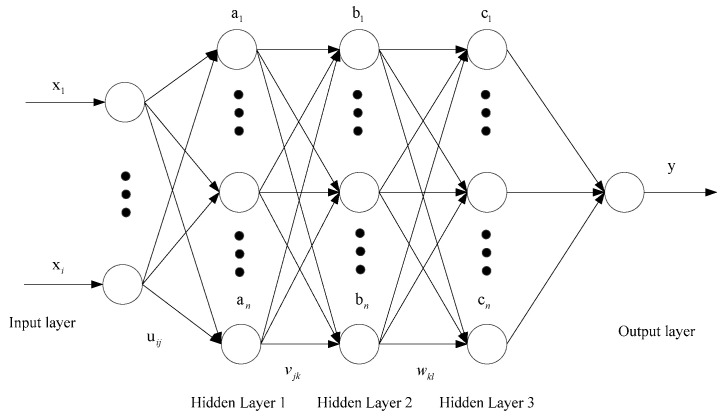
Structure diagram of three-layer BP neural network.

The first layer of the network predicts traffic data at the starting time point. In this layer network, the data transmitted by various sensors at the current moment are used as the input parameters of the model, and the initial flow value is predicted in real time. The second layer of the network predicts the parameters of the next time point. The traffic value predicted by the first layer of the network and other data from the same time point are taken as input data to predict the data at the next moment. The third layer of the network predicts the next time shift. All types of data predicted by the second layer of the network are input into the layer network model, and finally, the hydraulic cylinder displacement data at the next time point are obtained. The structure of the three-layer neural network is shown in Figure 5. The formula of the neural network is as follows:

(18) $$y=\Theta \left\{\sum_{k=1}^{n_{3}}w_{kl}\Theta \left[\sum_{j=1}^{n_{2}}v_{jk}\Theta \left(\sum_{i=1}^{n_{1}}u_{\mathrm{ij}}x_{i}+u_{0i}\right)+v_{0j}\right]+w_{0k}\right\}$$

Here, n is the number of nodes in the hidden layers; $x_{i}$ is the input vector; $u_{\mathrm{ij}}$ is the weight from $x_{i}$ to the first hidden layer neuron $a_{n}$; $v_{jk}$ is the weight of neuron $a_{n}$ from $b_{n}$ to the second hidden layer; $w_{kl}$ is the weight from $b_{n}$ to the third hidden layer neuron $c_{n}$; $u_{0i}$ is the bias of $a_{n}$; $v_{0j}$ is the offset of $b_{n}$; $w_{0k}$ is the bias of $c_{n}$; Θ is the activation function for each hidden layer; and y is the output vector.

The activation function for all neurons in the hidden layers of the neural network is designated as the sigmoid function:

(19) $$f\left(x\right)=\frac{1}{1+e^{-x}}$$

**Figure 6:**
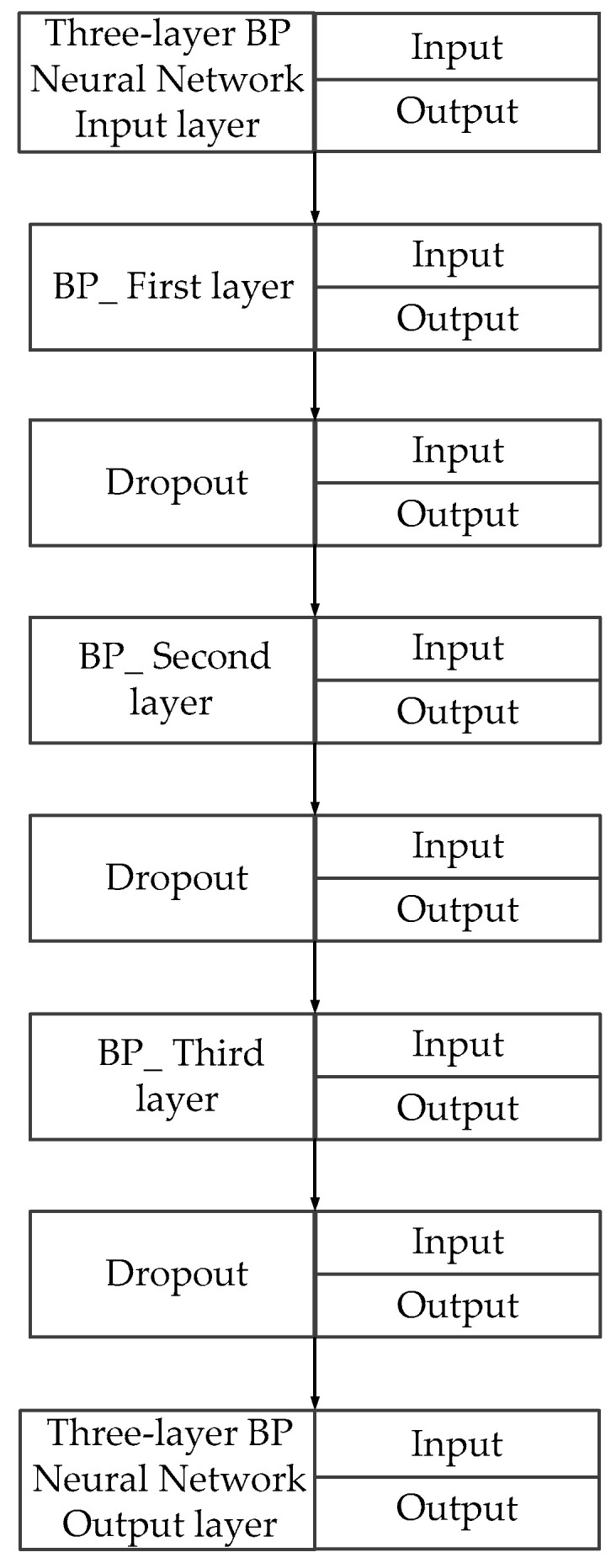
Internal logic of three-layer BP neural network architecture.

Figure 6 is a schematic diagram of the neural network architecture. Given the strong fitting capability of the three-layer neural network, to prevent the occurrence of “overfitting” after multiple training iterations [38], this study implemented dropout in each hidden layer to enhance the model’s generalization ability. Specifically, dropout involves actively deactivating a number of nodes in the multilayer perceptron, rendering them temporarily inactive [39].

The current moment is set to $t_{0}$ and the predicted next moment is set to $t_{1}$. The displacement of the piston rod of the hydraulic cylinder at time $t_{0}$ is $x_{0}$, the displacement of the piston rod of the hydraulic cylinder at time $t_{1}$ is $x_{1}$, the displacement difference of the piston rod of the hydraulic cylinder at the two moments is Δx, the time interval is Δt, and the piston area of the inner wall of the hydraulic cylinder is $S_{0}$; thus, the theoretical flow rate of the hydraulic cylinder at the current moment, temperature, and pressure is $Q_{L}$, and the calculation equation is as follows:

(20) $$Q_{L}=\frac{\Delta xS_{0}}{\Delta t}$$

The response time of a typical proportional control valve a and reversing valve is approximately 10 ms to 70 ms [40], so the total prediction time is set to 100 ms.

### 3.3. The First Layer of the BP Neural Network Model

The completed data need to be normalized in order to prevent gradient explosion in the training process [41]. The specific calculation method is shown in Equation (21):

(21) $$x^{\prime }=\frac{x-x_{\min}}{x_{\max}-x_{\min}}$$

The input values of the first layer of the BP neural network are the pressure p of the non-rod chamber, the temperature T of the non-rod chamber, the pressure difference Δp between the non-rod and rod end chambers, the displacement difference Δx, and the time difference Δt. The output value is the actual flow rate of the oil inlet $Q_{S}$.

In model training, 60% of the data in each dataset were selected as the training set and the remaining data were selected as the test set. Because the neural network model is prone to overfitting, the fitting effect on the training set was much better than that on the test set. Therefore, 20% of the data in the training set were selected as the verification set in the training process, and 10-fold cross-validation was adopted according to the amount of experimental data: 10-fold cross-validation can effectively prevent the occurrence of underfitting and overfitting, and so was carried out within the training and verification sets. The test set is never involved in cross-validation and is only used in the final evaluation of the model. The specific steps are as follows:

(1) The data should be randomly and evenly divided into 10 subsets. The data distribution of each subset should be consistent with the original data as much as possible. The data division should be randomized to avoid order deviation.
(2) Cross-validation rounds, training the model with the training set, and evaluating the model performance on the verification set.
(3) The validation results of each round are recorded, and the average value of 10 validation results is used as the generalization performance estimation of the model.

**Figure 7:**
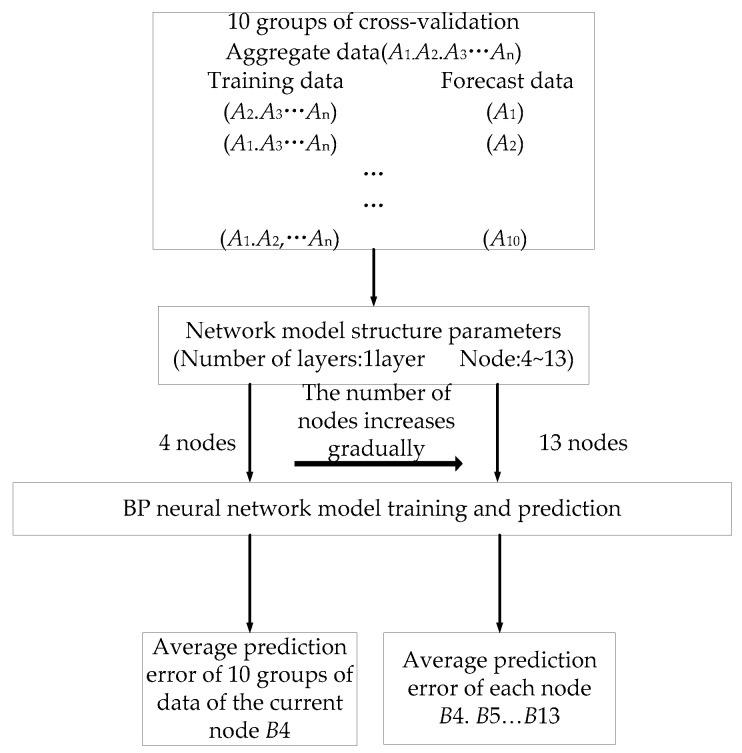
Schematic diagram of predictive performance of the evaluation network model.

The number of hidden layers is set to 1, and the range of nodes is calculated by using empirical equation $\sqrt{m+n}+A$, where m and n are the numbers of nodes in the input and output layers, respectively. The value range of A is 1 to 10 and the number of nodes in the hidden layer is 4 to 13. The process of evaluating the predictive performance of the network model is shown in Figure 7.

After data preprocessing, the maximum displacement at time $t_{0}$ is 239.746 mm and the minimum displacement is 6.104 mm. Supposing that the maximum allowable error data of prediction is $x_{m}$ and that the true value is $x_{n}$, the calculation equation is shown as Equation (22):

(22) $$\frac{x_{m}-6.104}{239.746-6.104}-\frac{x_{n}-6.104}{239.746-6.104}=\frac{x_{m}-x_{n}}{233.642}$$

The final displacement prediction error should be within 1 µm. The final iteration error normalization value is 0.233642. The maximum number of iterations is set to 2000, the learning rate is set to 0.03, and the initial weights and biases are set to random numbers from 0 to 1.

**Figure 8:**
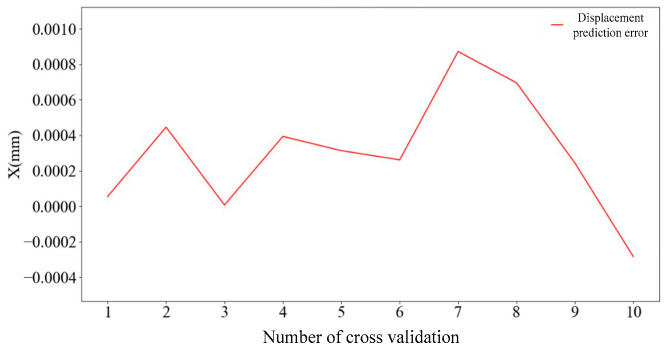
Average prediction error per node.

There are prediction errors in every cross-validation, and there are 10 average prediction errors after 10 cross-validations. Figure 8 shows the change curve of the average prediction error value corresponding to ten cross-validations of a single node.

**Figure 9:**
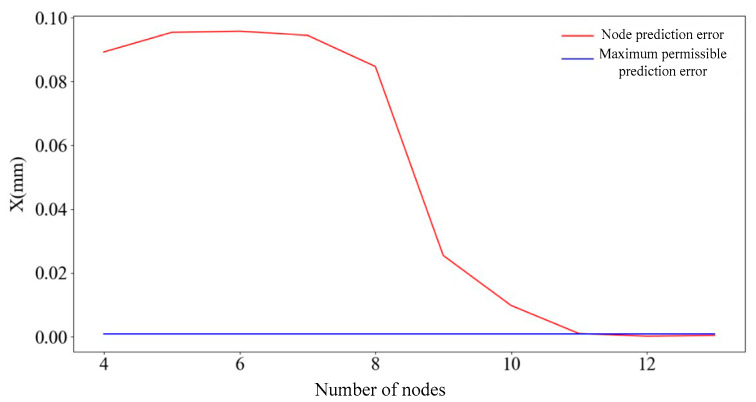
The corresponding error of each node of the neural network.

By cycling the above process 10 times, the average prediction error of 10-fold cross-validation corresponding to each node can be obtained, and the results are shown in Figure 8. As can be seen in Figure 9, when the numbers of nodes are 11, 12, and 13, the average prediction error is less than 0.001 mm. The number of nodes corresponding to the minimum prediction error is 12 and the error value is 0.4005699; therefore, the number of nodes selected for the first layer of the neural network is 12.

Some numerical errors are added to the part where the output parameters of the second and third layers of the BP neural network model coincide with the input parameters of the first layer of the BP neural network model, and the prediction accuracy of the second and third layers of the BP neural network model is judged based on the error accuracy of the output displacement.

This is because in the design of the three-layer BP neural network model, although dropout is introduced, it remains necessary to test whether the model relies on redundant information or multipath processing to correct errors. We evaluated the tolerance of subsequent network layers to input perturbations in order to determine whether they over-depended on specific patterns in the training data. By introducing artificial errors, the robustness, fault tolerance, and parameter sensitivity of the three-layer BP neural network model can be systematically assessed, providing a basis for model optimization. If the second and third layers maintain stable outputs despite input parameter errors, this demonstrates strong adaptability and validates the effectiveness of the hierarchical design. Conversely, if errors significantly degrade the output, further refinement of the neural network training strategy is required.

The error values are added in the range of ±0.1, ±0.01, and ±0.001, and the error values are added as a random function. The prediction verification cycle was carried out 100 times, the average of the difference between the predicted and actual data in each table was taken, and a data error scatterplot was output.

**Figure 10:**
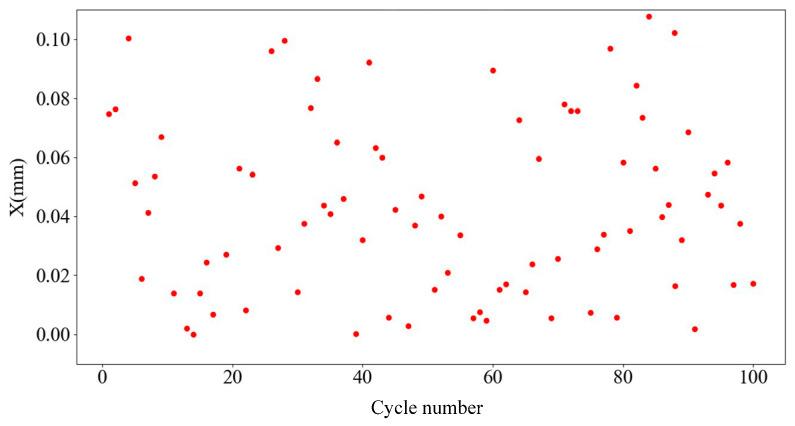
±0.1 Random error value.

**Figure 11:**
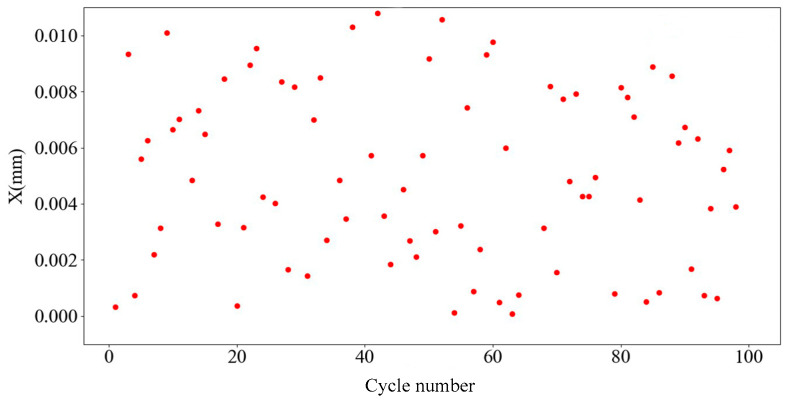
±0.01 Random error value.

**Figure 12:**
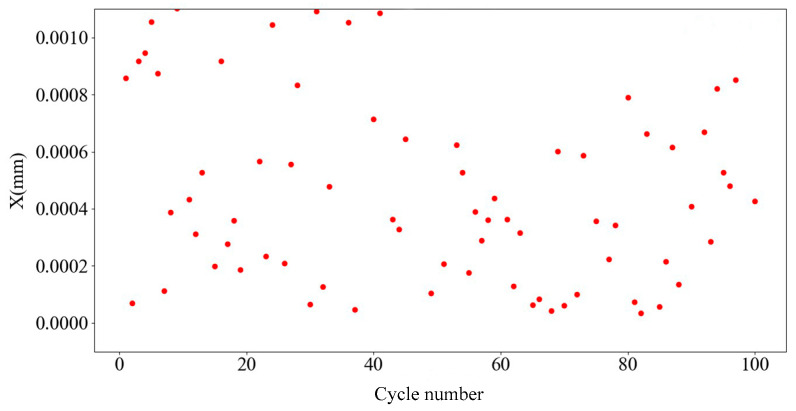
±0.001 Random error value.

Figure 10, Figure 11 and Figure 12 present scatterplots of the average predicted displacement error values of 100 cycles corresponding to random errors of ±0.1, ±0.01, and ±0.001 respectively. The average values of 100 points corresponding to random errors of ±0.1, ±0.01, and ±0.001 are 0.06780, 0.00727, and 0.00074, respectively. Therefore, the prediction error of the second and third layers of the BP neural network is ±0.001.

### 3.4. The Second Layer of the BP Neural Network Model

The training process of the second layer of the neural network model is analogous to that of the first layer of the neural network model. The difference is that the second layer of the hidden layer network model performs cycle training prediction for each node combination model.

The input value of the second layer of the BP neural network is the non-rod chamber pressure $p_{n}$ at time $t_{0}$, the rod end chamber pressure $p_{r}$, the pressure difference between the two chambers Δp, the temperature of the non-rod chamber T, the actual flow rate of the oil inlet $Q_{S}$, and time $t_{1}$. The output is the non-rod chamber pressure $p_{n}$ at time $t_{1}$, the rod end chamber pressure $p_{r}$, the pressure difference between the two chambers Δp, the temperature of the non-rod chamber T, and the actual flow rate of the oil inlet $Q_{S}$.

The combinations of nodes that meet the precision requirements for predicting the influencing factors, along with their corresponding mean prediction errors, are presented in Table 1. As can be observed, the node combinations (9, 10), (11, 13), and (12, 9) all satisfy the criteria. However, a model with fewer nodes is simpler and requires a shorter computational time. Therefore, the node combination (9, 10) is selected for the hidden layer of the second layer of the BP neural network model.

### 3.5. The Third Layer of the BP Neural Network Model

**Figure 13:**
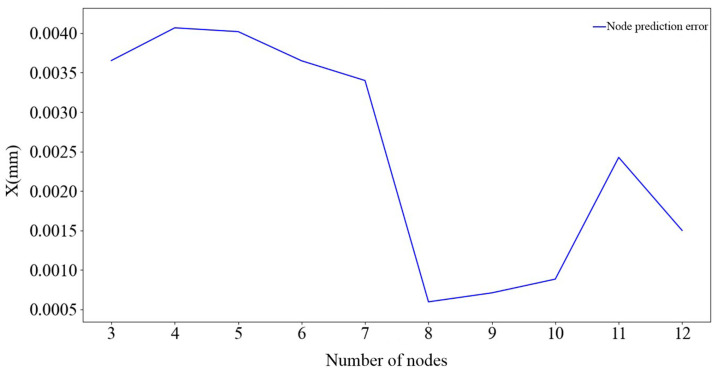
Prediction error corresponding to each node.

The input factors of the third layer of the BP neural network are the pressure $p_{n}$ of the non-rod chamber at time $t_{1}$, the pressure $p_{r}$ of the rod end chamber, the pressure difference between the two chambers Δp, the temperature T of the non-rod chamber, the actual flow rate of the oil inlet $Q_{S}$, the pressure difference $\Delta p_{1}$ of the non-rod chamber at time $t_{1}$ and time $t_{0}$, the displacement $x_{0}$ at time $t_{0}$, and time $t_{1}$. The predicted value is the piston rod displacement $x_{1}$ at time $t_{1}$. The network training process is consistent with that of the first and second layers of the BP neural networks. The node prediction error of the obtained neural network is shown in Figure 13.

As can be seen in Figure 13, when the number of nodes is 8, the prediction error is the smallest, 0.00059 mm, which is within the error range of 0.001. Therefore, the number of nodes of the third layer of the BP neural network is set to 8.

## 4. Prediction Model Validation and Optimization

To evaluate the accuracy of the model’s predictions, this study employed the mean absolute error (MAE), root mean square error (RMSE), and the coefficient of determination (R^2^), which are standard metrics for assessing the performance of predictive models. The computational formulas are as follows:

(23) $$MAE=\frac{1}{N}\sum_{i=1}^{N}\left|\left(y_{i}-{\hat{y}}_{i}\right)\right|$$

(24) $$RMSE=\sqrt{\frac{1}{N}\sum_{i=1}^{N}({\begin{array}{c} y_{i}-\hat{y_{i}}) \end{array}}^{2}}$$

(25) $$R^{2}=1-\frac{\sum_{i=n}^{N}({\begin{array}{c} y_{i}-{\hat{y}}_{i}) \end{array}}^{2}}{\sum_{i=n}^{N}({\begin{array}{c} y_{i}-{\bar{y}}_{i}) \end{array}}^{2}}$$

Here, N represents the total number of test data points, $y_{i}$ and $\hat{y_{i}}$ denote the actual and predicted values of the i data point, respectively, and $\bar{y}$ is the mean of the actual values.

**Figure 14:**
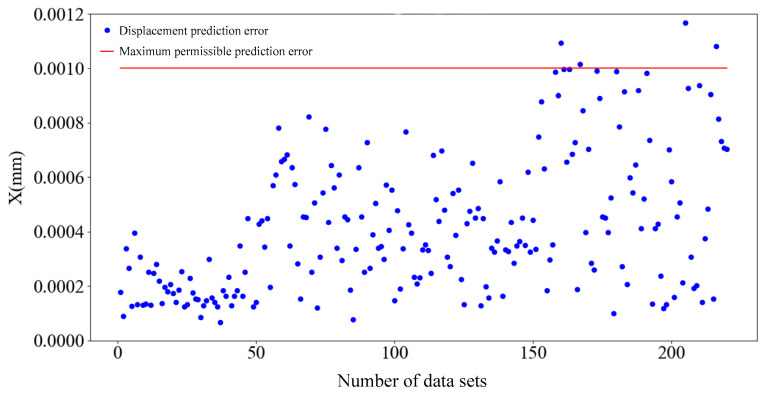
Scatterplot of test set displacement prediction error.

The experimental data were input into both the BP neural network prediction model and the three-layer BP neural network prediction model. As can be discerned from the predictive performance evaluation metrics presented in Table 2, the MAE and RMSE values of the three-layer BP neural network prediction model are both lower than those of the BP neural network prediction model. In a comparative assessment of R^2^, the three-layer BP neural network prediction model exhibits a closer proximity to 1, indicating a superior fit and a closer alignment with the actual displacement values. Consequently, an analysis of Table 2 substantiates that the three-layer BP neural network prediction model achieves higher predictive accuracy. A scatterplot illustrating the displacement prediction errors of the three-layer BP neural network predictive model is presented in Figure 14.

When the number of datasets is within 50, the error of the prediction model is lower and more concentrated. When the number of datasets is between 50 and 100, the error of the prediction model begins to increase, but the displacement prediction error is concentrated in the range of 0–0.0009 mm. When the number of datasets is greater than 150, the prediction error begins to rapidly increase, the prediction data become increasingly more discrete, and the prediction error exceeds the maximum allowable error: the maximum error even reaches 1.1736 µm.

The maximum operation time for each group is collected using the loop function, as shown in Table 3.

As can be observed in Table 3, the computational time of the prediction model is the longest at the initial time point, with a duration of 60.58 ms. The calculation time of the other prediction time is basically the same, being in accordance with the calculation rule of the model. However, the maximum time that can be reserved for the sum of the reversing time of the reversing valve in the hydraulic system and the buffer time of the oil in the hydraulic cylinder is only 44.07 ms.

When 50 and 150 datasets are taken as the dividing lines, the prediction accuracy of the model clearly decreases. It can be seen in Table 4 that the median of the test factors increases with the increase in the number of datasets, and the medians of the flow, temperature, and pressure of the experimental data after 150 datasets are basically higher than those of other data. Therefore, the change in the average error value of the prediction model is related to the size of the input factor value.

In the continuous operation of a hydraulic cylinder, under high-flow and high-pressure conditions, impurities will inevitably occur in the oil, and the high-frequency vibration [42] generated by the motor due to the high load will also affect the displacement of the hydraulic cylinder and the physical and chemical properties of the oil. Furthermore, these external factors will affect the prediction accuracy of the model. Therefore, if the physical value of the experimental factors increases to a certain extent, the prediction accuracy of the model will be affected.

**Figure 15:**
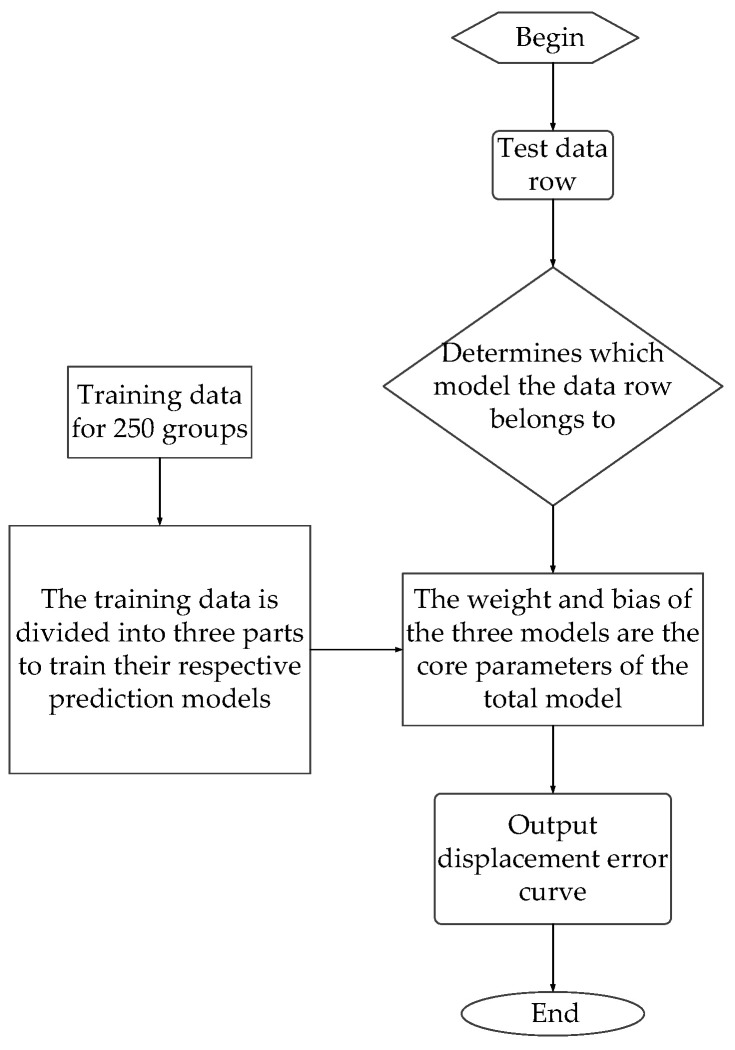
Predictive model optimization scheme training process.

The prediction model was optimized according to Figure 15. Firstly, three sets of weights and biases were obtained through training. Each of these three sets of weights and biases was consistent with the dimensions of the weights and biases in the original prediction model. After the prediction model is completed, when the test data are used for testing, various pressure, temperature, flow and other parameters of the data line should be logically judged to determine which type of input data belongs to the model. Then, it should be input into the corresponding weight and bias function, and output errors will be obtained after the main program is run.

By modulating the weights, the precision and computational efficiency of the algorithm can be altered, thereby aiding in the pursuit of the optimal solution. A novel approach is proposed, which introduces a nonlinear adaptive decreasing weight mechanism to enhance the optimization accuracy of the algorithm. The corresponding formula is as follows:

(26) $$\omega (t)=\omega_{\max}-(\omega_{\max}-\omega_{\min})/(1+e^{-5t/T_{\max}})$$

Here, ω represents the weighting coefficient, $\omega_{\max}$ denotes the maximum value of the weighting coefficient, and $\omega_{\min}$ signifies the minimum value of the weighting coefficient.

**Figure 16:**
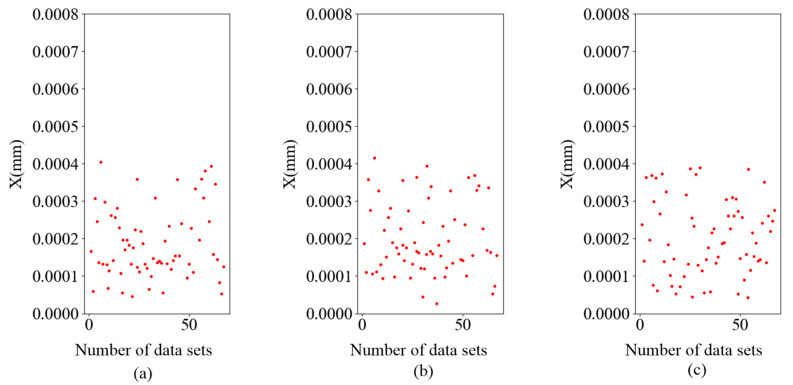
(**a**) Model 1 predicts the scatterplot of displacement errors, (**b**) model 2 predicts the scatterplot of displacement errors, and (**c**) model 3 predicts the scatterplot of displacement errors.

Next, 66 sets of data were taken from the test sets of each of the three prediction models and input into the prediction model. After training the three-layer BP neural network, the weight and bias were obtained for each of the three prediction models. The three subgraphs in Figure 16 present the displacement error values output from the three models.

As can be seen in Figure 16, the average error point distribution of each table is below 4.5 × 10^−4^ mm, and the average error of data error points in the three subgraphs is 1.8909 × 10^−4^ mm, 1.8911 × 10^−4^ mm, and 2.024 × 10^−4^ mm, respectively. The accuracy of the three prediction models is within the expected range, and they are more accurate than the prediction model before optimization. Finally, the weight and bias of the updated three prediction models were output.

**Figure 17:**
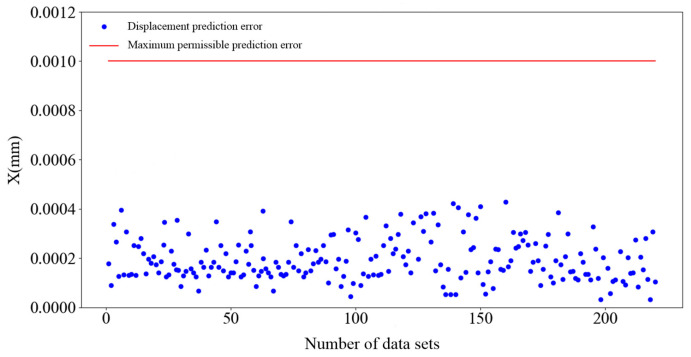
Scatterplot of the displacement error predicted by the optimized prediction model.

Figure 17 shows the displacement prediction errors of each group output by the optimized prediction model. A comparison of Figure 14 and Figure 17 shows that the prediction accuracy of the optimized prediction model for different data samples is significantly improved, and the average prediction error of 250 datasets is 0.1691 μm. The maximum displacement prediction error in the test set is 0.5491 μm.

The maximum operation time for each group is collected using the loop function, as shown in Table 5.

As can be seen in Table 5, the maximum operation time of the optimized prediction model is only 27.82 ms. The maximum time that can be reserved for the sum of the reversing time of the reversing valve in the hydraulic system and the oil buffer time in the hydraulic cylinder is 74.57 ms.

## 5. Conclusions

In this study, we propose a predictive model based on a three-layer BP neural network. The first layer of the BP neural network takes the data transmitted by various sensors at the current moment as input parameters to predict the initial flow value in real time. The second layer of the BP neural network utilizes the flow value predicted by the first neural network, along with other concurrent data, as input to forecast the data of the next time step. The third layer of the BP neural network inputs the various data predicted by the second layer into its network model to obtain the hydraulic cylinder displacement data of the subsequent time step. The initial experimental results exhibited significant errors. By proposing a nonlinear adaptive decreasing weight mechanism to enhance the optimization accuracy of the algorithm, the search for optimal solutions is facilitated, ultimately leading to algorithm optimization.

The experimental results demonstrate that the optimized hydraulic cylinder displacement prediction model achieves significant improvements in precision, computational efficiency, and system responsiveness. The maximum predicted displacement error is reduced to 0.5491 μm, thereby improving the accuracy by approximately 53% compared with the original prediction error of 1.1736 μm. This enhancement translates directly into a higher precision of displacement control. The maximum run time of the predictive model is reduced to 27.82 ms, which improves the real-time processing capability of the model in a dynamic environment. The maximum allowable time allocated for reversing valve switching and hydraulic oil buffering in the system is extended by nearly 1.7 times. A greater time margin is provided for the system actuator to respond to predicted displacement changes. The nonlinear adaptive weight mechanism effectively addresses nonlinearities and transient dynamics in hydraulic systems, minimizing the cumulative errors caused by fluid compressibility and leakage. By refining the weight optimization strategy, redundant computational paths are eliminated, accelerating convergence during neural network training.

The proposed prediction model demonstrates robust adaptability to hydraulic cylinder control under heterogeneous types, operational conditions, and service durations by dynamically adjusting its weights and biases through the collection of diversified experimental datasets. Furthermore, an in-depth analysis of the input–output curves generated by the model enables an accurate assessment of the hydraulic cylinder operational status, as well as the reliability and wear progression of internal components. The experimental findings presented in this study hold both theoretical and practical significance for advancing cost-effective and high-precision automated control strategies in hydraulic cylinder systems. However, actual leakage scenarios may involve turbulent flow conditions, including rapid pressure fluctuations, large-scale leakage defects, high-viscosity fluids, or significant pressure differentials. Under these fault conditions, the prediction model proposed in this study exhibits certain limitations. It is also worth considering how the system responds to adhesion and sliding conditions. Future improvements may focus on optimizing the neural network architecture and hyperparameters or integrating advanced neural network optimizers to further elevate the model’s predictive accuracy.

In the future, hydraulic cylinder displacement sensors are anticipated to evolve towards higher precision, enhanced environmental adaptability, and reduced costs. Concurrently, with the integration of artificial intelligence and big data technologies, these sensors are expected to exhibit advanced capabilities in data analysis and predictive functionalities, thereby providing robust support for the intelligentization of hydraulic systems.

## Figures and Tables

**Table 1:** Nodes and average errors that meet the prediction accuracy requirements of five influencing factors.

Node Combination	Average Error of *p* (10^−4^)	Average Error of *p*_1_ (10^−4^)	Average Error of ∆p (10^−4^)	Average Error of *T* (10^−4^)	Average Error of *Q*_S_ (10^−4^)
(6, 13)	65.7389	5.436	3.786	4.3389	46.963
(7, 5)	65.963	8.739	22.928	2.28	153.36
(7, 6)	48.968	13.328	48.6445	8.756	12.65
(7, 7)	2.89661	6.536	6.86889	2.948	40.5
(8, 12)	12.6895	8.6942	23.6552	2.6354	23.6652
(9, 10)	6.89	5.486	3.458	2.2533	8.714
(11, 11)	6.04253	12.3666	2.3665	5.146	9.3645
(11, 11)	5.51733	2.2334	3.889	3.658	5.1526
(12, 9)	5.66	2.336	4.365	1.336	6.5361

**Table 2:** Comparison of each evaluation index of prediction model.

Prediction Model	Evaluation Index		
	MAE	RMSE	R^2^
BP neural network	1.402	1.587	0.909
Three-layer BP neural network	0.912	1.076	0.935

**Table 3:** Maximum operation time of data prediction model.

Interval of the Dataset	Maximum Operation Time for Each Group of Prediction Model (ms)				
(0–50)	60.58	60.58	60.56	60.57	60.56
(51–100)	56.06	55.96	55.97	55.97	55.98
(101–150)	55.99	55.97	55.95	55.93	55.95
(151–200)	55.95	55.97	55.97	55.95	55.94
(210–250)	55.95	55.96	55.93	55.95	55.94

**Table 4:** The median of each input factor of the sensor.

Number of Datasets	Non-Rod Chamber Pressure (MPa)	Rod End Chamber Pressure (MPa)	Temperature (°C)	Flow Rate (L/min)
<50	2.6	0.15	24	2.3
>50 and <150	6.6	0.18	40	4.1
>150	8	0.26	46	5.3

**Table 5:** Maximum operation time of the optimized data prediction model.

Interval of the Dataset	Maximum Operation Time for Each Group of Prediction Model (ms)				
(0–50)	27.82	27.78	27.75	27.73	27.73
(51–100)	25.48	25.47	25.48	25.45	25.43
(101–150)	25.47	25.49	25.47	25.46	25.45
(151–200)	25.45	25.44	25.45	25.48	25.43
(210–250)	25.45	25.46	25.49	25.45	25.44

## Data Availability

According to the confidentiality of the funding project, the codes and data supporting the survey results of this paper are not disclosed at present because the research has not been completed. You can request a copy from the author at zyr894416121@163.com.

## Abbreviations

The following abbreviations are used in this manuscript:

BP	backpropagation
ABSC	adaptive backstepping control
PID	proportional integral derivative
LMS	least mean square
**Variable**	**Meaning**
β	compressibility
E	bulk elastic modulus
$Q_{0}$	oil flow loss of the non-rod chamber of the hydraulic cylinder
$\alpha_{V}$	thermal expansibility
$\mu_{t}$	dynamic viscosity when the temperature is T °C
$\mu_{0}$	$\mathrm{dynamic}\;\mathrm{viscosity}\;\mathrm{when}\;\mathrm{the}\;\mathrm{temperature}\;\mathrm{is}\;T_{0}\;$°C
$\mu_{p}$	viscosity when the pressure is p
$\mu_{1}$	viscosity when the pressure is 0.1 MPa
$\mu_{B}$	dynamic viscosity of oil when the proportion of mixed air is B
$\mu_{2}$	dynamic viscosity of oil without mixing air, which is a fixed value
$\mu_{C}$	dynamic viscosity of the oil with pressure p, temperature T, and mixed gas amount B
$\mu_{3}$	$\mathrm{dynamic}\;\mathrm{viscosity}\;\mathrm{when}\;\mathrm{the}\;\mathrm{pressure}\;\mathrm{is}\;101.03\;\mathrm{KPa},\;\mathrm{the}\;\mathrm{temperature}\;\mathrm{is}\;T_{0}$, and no gas is mixed
$K_{a}$	coefficient factor
$q_{p}$	amount of oil leakage when considering the influence of pressure
$Q_{1}$	$\mathrm{internal}\;\mathrm{leakage}\;\mathrm{flow}\;\mathrm{of}\;\mathrm{the}\;\mathrm{hydraulic}\;\mathrm{cylinder}\;\mathrm{when}\;\mathrm{the}\;\mathrm{temperature}\;\mathrm{is}\;T_{t}$, and the mixed air concentration is B
$Q_{2}$	external leakage flow loss of a hydraulic cylinder
$Q_{L}$	theoretical flow rate of the oil inlet
$Q_{S}$	actual flow rate of the oil inlet
